# Characterization of Metabolically Quiescent *Leishmania* Parasites in Murine Lesions Using Heavy Water Labeling

**DOI:** 10.1371/journal.ppat.1004683

**Published:** 2015-02-25

**Authors:** Joachim Kloehn, Eleanor C. Saunders, Sean O’Callaghan, Michael J. Dagley, Malcolm J. McConville

**Affiliations:** 1 Department of Biochemistry and Molecular Biology, Bio21 Institute of Molecular Science and Biotechnology, University of Melbourne, Parkville, Victoria, Australia; 2 Metabolomics Australia, Bio21 Institute of Molecular Science and Biotechnology, University of Melbourne, Parkville, Victoria, Australia; National Institute of Health, UNITED STATES

## Abstract

Information on the growth rate and metabolism of microbial pathogens that cause long-term chronic infections is limited, reflecting the absence of suitable tools for measuring these parameters *in vivo*. Here, we have measured the replication and physiological state of *Leishmania mexicana* parasites in murine inflammatory lesions using ^2^H_2_O labeling. Infected BALB/c mice were labeled with ^2^H_2_O for up to 4 months, and the turnover of parasite DNA, RNA, protein and membrane lipids estimated from the rate of deuterium enrichment in constituent pentose sugars, amino acids, and fatty acids, respectively. We show that the replication rate of parasite stages in these tissues is very slow (doubling time of ~12 days), but remarkably constant throughout lesion development. Lesion parasites also exhibit markedly lower rates of RNA synthesis, protein turnover and membrane lipid synthesis than parasite stages isolated from *ex vivo* infected macrophages or cultured *in vitro*, suggesting that formation of lesions induces parasites to enter a semi-quiescent physiological state. Significantly, the determined parasite growth rate accounts for the overall increase in parasite burden indicating that parasite death and turnover of infected host cells in these lesions is minimal. We propose that the *Leishmania* response to lesion formation is an important adaptive strategy that minimizes macrophage activation, providing a permissive environment that supports progressive expansion of parasite burden. This labeling approach can be used to measure the dynamics of other host-microbe interactions *in situ*.

## Author Summary

Microbial pathogens can adapt to changing conditions in their hosts by switching between different growth and physiological states. However, current methods for measuring microbial physiology *in vivo* are limited, hampering detailed dissection of host-pathogen interactions. Here we have used heavy water labeling to measure the growth rate and physiological state of *Leishmania* parasites in murine lesions. Based on the rate of *in situ* labeling of parasite DNA, RNA, protein, and lipids, we show that the growth rate of intracellular parasite stages is very slow, and that these stages enter a semi-quiescent state characterized by very low rates of RNA, protein, and membrane turnover. These changes in parasite growth and physiology are more pronounced than in *in vitro* differentiated parasites, suggesting that they are induced in part by the lesion environment. Despite their slow growth, the parasite burden in these lesions progressively increases as a result of low rates of parasite death and host cell turnover. We propose that these changes in *Leishmania* growth and physiology contribute to the development of a relatively benign tissue environment that is permissive for long term parasite expansion. This approach is suitable for studying the dynamics of other host-pathogen systems.

## Introduction

A number of medically important bacterial, fungal and protozoan pathogens are associated with persistent chronic infections that can reactivate to cause acute disease long after initial infection [1–5]. With few exceptions [6], very little is known about the growth rate or physiological state of these pathogens during chronic stages of infection, reflecting limitations in current methods for measuring microbial growth *in situ*. This information is crucial for modeling host-pathogen dynamics and developing therapies that target these stages.

*Leishmania* spp are protozoan parasites that are associated with long-term chronic infections, as well as acute disease, ranging from self-healing cutaneous lesions to fatal visceral infections, in millions of people worldwide [7]. Infection is initiated by flagellated promastigote stages that are injected into the skin by a sandfly vector. Following their uptake by macrophages and other phagocytic cells, promastigotes differentiate to aflagellate amastigotes that proliferate in the phagolysosome compartment of these host cells [8,9]. A hallmark of all *Leishmania* infections is the formation of localized tissue lesions or granulomas composed primarily of infected and uninfected macrophages, at the site of the sandfly bite or in distal tissues such as the liver and spleen [10–12]. Depending on the *Leishmania* species involved and host genetics, lesion formation can be associated with immune control (but usually not eradication of the parasite) or parasite expansion and systemic infection. In murine models of infection, host resistance is associated with the development of a T-helper type 1 response, while lesion development occurs in susceptible animals that mount a T-helper type 2 response [13,14]. In contrast to our understanding of the host immune responses that underlie these different outcomes, very little is known about the growth rate or physiological state of *Leishmania* in these tissues. Transgenic parasite lines expressing luciferase or different fluorescent proteins have been developed and used to visualize parasite dynamics *in vivo* [15–18]. However, these approaches only provide a measure of net changes in parasite burden, which are determined by rates of parasite death and migration from infected tissues, as well as rates of replication. Furthermore, attempts to infer the physiological status of lesion amastigotes from transcriptomic and proteomic analyses have been hampered by the absence of conventional gene-specific transcriptional control in these parasites and the paucity of coordinated changes in the abundances of individual mRNA and proteins in different insect and mammalian-infective stages [19,20].

**Fig 1:**
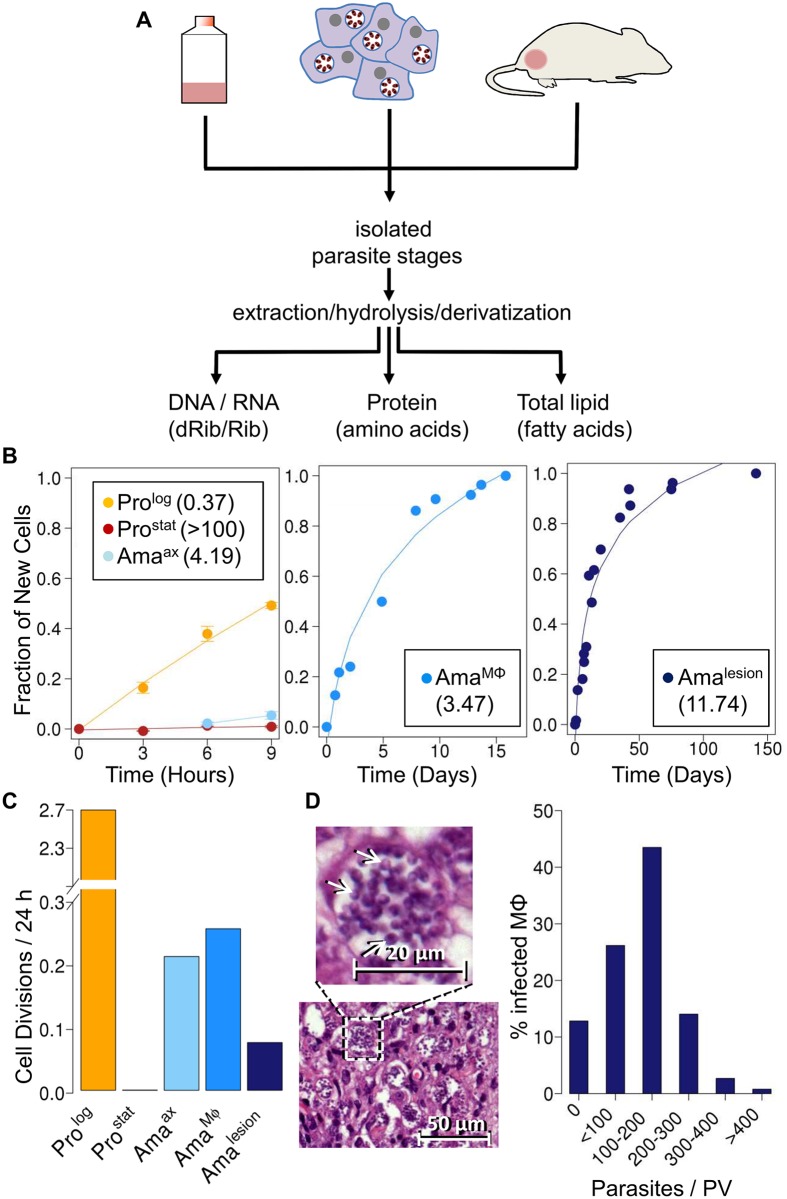
Stage-specific changes in *Leishmania* growth rates. **A**. Schematic overview of ^2^H_2_O labeling protocol. Parasite stages were cultivated axenically in the presence 5% ^2^H_2_O, or isolated from infected macrophages or BALB/c lesion incubated or infused with ^2^H_2_O (final concentration 5%). Parasite stages were harvested at multiple time points and extracts containing total DNA/RNA, or total proteins and lipids generated from purified parasite fraction. Levels of deuterium enrichment in constituent dRib/Rib, amino acids and fatty acids were subsequently quantitated by GC-MS. **B**. Kinetics of ^2^H-labeling of DNA dRib in cultured promastigotes (Pro^log^, Pro^stat^) and amastigotes (Ama^axenic^), and in amastigotes isolated from macrophages (Ama^Mø^) and murine lesions (Ama^lesion^). The fraction of new cells (Y-axis) was calculated from the level of ^2^H-enrichment in dRib relative to maximum labeling observed in each parasite stage after long term (equilibrium) labeling. Calculated doubling times for each stage are shown in inset boxes. **C**. Comparative growth rates of different *Leishmania* stages, calculated from ^2^H-enrichment in dRib. **D**. Section of stained cutaneous lesion (with detail in insert) and calculated range of parasite numbers/phagolysosome. Abbreviations: dRib; deoxyribose, Rib; ribose.

In this study we introduce the use of ^2^H_2_O labeling to measure *Leishmania* growth rate and metabolic activity in murine inflammatory lesions. In the presence of ^2^H_2_O, cells stably incorporate deuterium into a wide range of metabolites, which are subsequently incorporated into cellular macromolecules, providing a quantitative read-out of global rates of DNA replication, transcription, protein turnover and membrane lipid biosynthesis (S1 Fig., Fig. 1A)[21,22]. ^2^H_2_O is easily and safely administered to animals for periods of weeks to months and rapidly equilibrates across all tissues [23], making it suitable for measurement of slowly growing microbial populations in infected tissues. Using this approach, we show that amastigotes exhibit a constant, but very slow growth rate, in non-healing lesions and appear to enter into a distinct semi-quiescent metabolic state characterized by low rates of transcription and protein turnover. This quiescent state is distinct from that measured in non-dividing (insect) promastigote stages and may represent an adaptive response to a growth-restrictive intracellular microenvironment in granulomas. Using this approach we also identified parasite-specific metabolic pathways, such as polyunsaturated fatty acid biosynthesis that are up-regulated *in situ*. This approach has provided the first global analysis of the physiological state of the major mammalian-infective stage of *Leishmania* and is generally applicable to studying the *in vivo* growth and physiology of other microbial pathogens.

## Results

### Measurement of *L*. *mexicana* amastigote replication rates *in vivo*

As previously reported [24], cultivation of *L. mexicana* promastigotes stages in standard medium containing 5% ^2^H_2_O, leads to the deuterium-labeling of the deoxyribose (dRib) moiety of DNA (Fig. 1B). The labeling of dRib occurs as a result of gluconeogenesis, various sugar phosphate isomerization/epimerization reactions, the pentose phosphate pathway and ribonucleotide reductase (S1 Fig.), and the rate of incorporation of deuterium into promastigote DNA was growth-dependent. Specifically, the rate of labeling of exponentially growing promastigotes (Pro^log^) indicated a doubling time of 9 hours, concordant with cell counts, while no incorporation was observed in the DNA of non-dividing promastigotes (Pro^stat^) (Fig. 1B). Significantly, labeling of the deoxyribose moiety of DNA was not affected by supplementation of the culture medium with ribose or a range of nucleosides and nucleotides to the culture medium (S2 Fig.), indicating that *de novo* synthesis of ribose/dRib is not affected by salvage pathways. Pro^stat^ were induced to differentiate to axenic amastigotes (Ama^axenic^) by acidification of the medium and cultivation at elevated temperature. Following differentiation, Ama^axenic^ exhibited a doubling time of 4.2 days, substantially slower than Pro^log^ (Fig. 1B, C). These data support the notion that amastigote differentiation is associated with activation of the parasite stress responses and a reduction in maximum growth rate, independent of exogenous nutrient levels [24].

Having shown that ^2^H_2_O labeling can be used to quantitate rates of parasite replication; we extended this approach to directly measure amastigote proliferation in murine tissue lesions. BALB/c mice were infected with *L. mexicana* parasites and subsequently labeled with ^2^H_2_O following the appearance of cutaneous lesions. A constant level of 5% ^2^H_2_O in the body water was established by providing mice with a bolus of 100% ^2^H_2_O and subsequent inclusion of 9% ^2^H_2_O in the drinking water for up to several months (S3 Fig.). Mice were culled at various time points and lesion amastigotes (Ama^lesion^) isolated from infected tissues. Histological examination showed that these lesions primarily comprised heavily infected host cells (with parasites in large communal vacuoles) and no detectable necrosis (S4 Fig.). Ama^lesion^ purified from these tissues were free of intact host cells or nuclei, as determined by DAPI staining, and were further treated with DNAse to remove any extracellular host DNA released during tissue disruption (S5 Fig.). Contamination of parasite DNA with host DNA was estimated to be less than 20% as determined by direct quantitation of DNA (S5 Fig.). Deuterium enrichment in parasite DNA-dRib, increased with time, reaching a maximum 15% enrichment (EM_1_; excess molar fraction of M_1_) after about 40 days of labeling (Fig. 1B). Based on the rate of enrichment, Ama^lesion^ were found to have a remarkably constant doubling time of ~12 days, irrespective of the age of the lesion when the ^2^H_2_O labeling was initiated (ranging from 4 weeks to 4 months post-infection) (Fig. 1B,C). This growth rate is 32-fold slower than the maximum growth rate of promastigotes stages and approximately 4-fold slower than that measured for Ama^axenic^ (Fig. 1C), indicating that parasite growth in the granuloma microenvironment is highly constrained.

To investigate whether the slow growth of lesion amastigotes reflected growth-limiting conditions in the phagolysosome of infected macrophages, we measured amastigote replication in J774 macrophages. J774 macrophages were infected with *L. mexicana* promastigotes and internalized parasites allowed to differentiate to amastigotes, before cultures were incubated in the presence of 5% ^2^H_2_O. Infected macrophages were labeled for up to 20 days and amastigotes (Ama^Mø^) isolated at various time points. Intracellular Ama^Mø^ were found to have a doubling time of 4 days (Fig. 1B), comparable to that of Ama^axenic^ and substantially faster than observed in Ama^lesion^ (Fig. 1B). These results suggest that the phagolysosomal compartment of non-activated macrophages is not growth limiting for amastigotes *per se*, and that additional factors in the lesion environment constrain parasite growth and/or induce a slow growth phenotype.

Quantitative analysis of hematoxylin and eosin (H&E) stained sections of BALB/c lesions harvested at day 85 post-infection showed that the majority of granuloma macrophages harbored between 100–200 amastigotes/phagolysosome (Fig. 1D). These values are in close agreement with parasite vacuole densities calculated assuming; (i) that each vacuole was established by a single parasite invasion event, (ii) a constant amastigote doubling time of 12 days and (iii) zero parasite death (120 parasites/vacuole). The presence of macrophages with fewer than 100 amastigotes/phagolysosome (28% of all infected macrophages) may reflect influx of uninfected macrophages that have been infected for a shorter period of time, or a subpopulation of slower growing parasites. On the other hand, the presence of small number of hyper-infected macrophages (up to 400 amastigotes/phagolysosome) may reflect a subpopulation of amastigotes with a faster growth rate and/or the uptake of multiple promastigotes/amastigotes during the initial infection. Overall, these analyses indicate that parasite and host cell turnover in lesions is minimal and that a significant majority of the granuloma host cells were infected very early after injection of parasites.

### Measurement of RNA and protein turnover in lesion amastigotes

**Fig 2:**
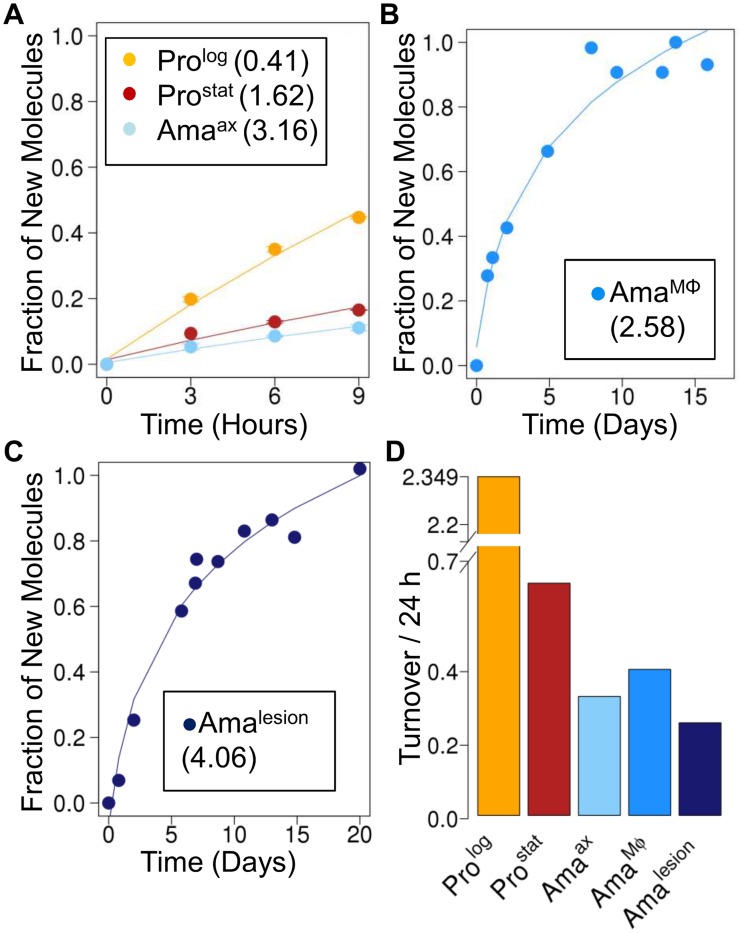
Rates of RNA turnover in cultured and intracellular *Leishmania* stages. Kinetics of ^2^H-labeling of RNA ribose in (**A**) cultured parasite stages (Pro^log^, Pro^stat^, Ama^axenic^) (**B**) amastigotes isolated from infected J774 macrophages (Ama^Mø^) and (**C**) amastigotes isolated from BALB/c lesions (Ama^lesion^). The fraction of new molecules (Y-axis) was calculated from the level of ^2^H-enrichment in Rib relative to maximum labeling observed in each parasite stage after long term labeling. Inset boxes shows estimated RNA turnover (t_1/2_ in days) in each stage. **D**. Comparative rates of RNA turnover in different *Leishmania* developmental stages.

To further define the physiological state of Ama^lesion^, we assessed the rate of incorporation of deuterium into the ribosyl moiety of RNA and proteinogenic amino acids in different parasite stages. Measurement of deuterium enrichment in the RNA ribosyl moiety primarily reflects ribosome biosynthesis, one of the most energy intensive processes in the cell [25] and is thus a measure of the metabolic state of a cell. As expected, Pro^log^ exhibited the highest rate of RNA turnover, comparable to the rate observed for DNA synthesis (Fig. 2A). Appreciable levels of RNA turnover were also observed in Pro^stat^, confirming that this non-dividing stage remains transcriptionally and metabolically active (Fig. 2A). Strikingly, all three amastigote stages (Ama^axenic^, Ama^Mø^ and Ama^lesion^) exhibited lower rates of RNA turnover than Pro^stat^, providing direct evidence that amastigote differentiation is linked to a general shut-down of energy intensive processes (Fig. 2A-C).

**Fig 3:**
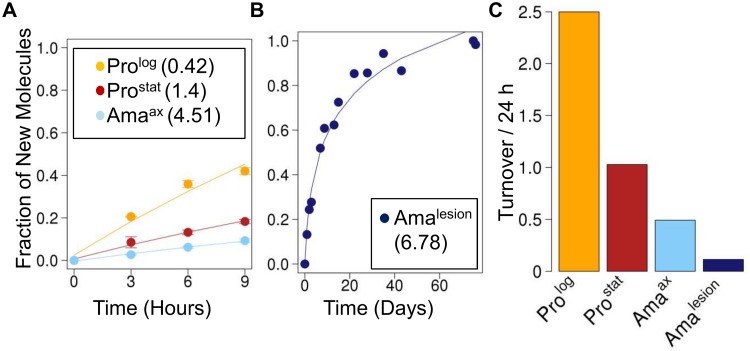
Rates of protein turnover in cultured and intracellular *Leishmania* stages. Parasite stages were ^2^H_2_O-labeled in culture or *in situ* in infected BALB/c mice and harvested at the indicated time points. Kinetics of ^2^H-labeling of proteinogenic alanine in (**A**) cultured parasite stages (Pro^log^, Pro^stat^, Ama^axenic^) and (**B**) amastigotes isolated from BALB/c lesion (Ama^lesion^). The fraction of new molecules (Y-axis) was calculated from the level of ^2^H-enrichment in alanine relative to maximum labeling observed in each parasite stage after long term labeling. Inset boxes in A and B show turnover (t_1/2_) in days. **C**. Comparative rates of protein turnover in different *Leishmania* developmental stages. Note that similar estimates of protein turnover were obtained by measuring deuterium incorporation into other proteinogenic amino acids (S6 Fig.).

The rate of protein synthesis/turnover provides another proxy for the metabolic state of a cell. As expected, deuterium was incorporated into a range of *Leishmania* proteinogenic amino acids via different transamination reactions and pathways of *de novo* biosynthesis [8,26] (S6A Fig.). While maximum levels of deuterium enrichment in different amino acids varied, with highest levels of enrichment in alanine (11% EM_1_) and glutamate (9% EM_1_), similar rates of protein turnover were calculated after normalization to maximum labeling, regardless of the amino acid used (S6B Fig.). Both dividing and non-dividing promastigote stages exhibited higher rates of protein synthesis than Ama^axenic^ and Ama^lesion^ (Fig. 3A-C). Interestingly, while the turnover of protein in Pro^log^ and Ama^axenic^ stages occurred at approximately the same rate as DNA replication, protein turnover in Ama^lesion^ occurred at nearly twice the rate of DNA synthesis. Thus although Ama^lesion^ stages have the lowest absolute rates of protein turnover, the cellular proteome is turned over more times per cell division cycle than in other stages.

### *Leishmania* amastigotes also exhibit reduced membrane turnover *in vivo*, but are dependent on *de novo* synthesis of key fatty acids

**Fig 4:**
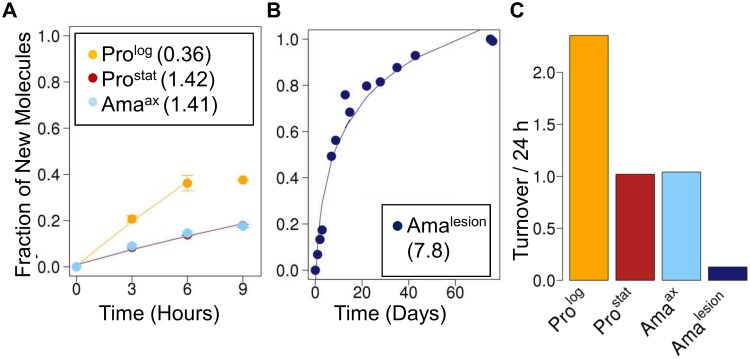
Stage-specific changes in fatty acid synthesis. Parasite stages were ^2^H_2_O-labeled in culture or *in situ* in infected BALB/c mice and harvested at the indicated time points. Kinetics of ^2^H-labeling of the major fatty acid, C18:0 (stearic acid) in (**A**) cultured parasite stages (Pro^log^, Pro^stat^, Ama^axenic^) and (**B**) amastigotes isolated from BALB/c lesion (Ama^lesion^). The fraction of new molecules (Y-axis) was calculated from the level of ^2^H-enrichment in stearate relative to maximum labeling observed in each parasite stage after long term labeling. Inset boxes show turnover (t_1/2_) in days. **C**. Comparative rates of stearic acid turnover in different *Leishmania* developmental stages.

The turnover of membrane phospholipids is intimately linked to cell division, organelle biogenesis and dynamic cellular functions, such as secretion and endocytosis. To investigate whether membrane turnover is reduced in *Leishmania* amastigotes we measured global rates of fatty acid turnover in different cultured and lesion-derived stages. Fatty acids are primarily incorporated into phospholipids, with little incorporation into other lipids (such as triacylglycerol), providing a direct measure of membrane biogenesis. As with proteinogenic amino acids, the extent to which ^2^H-is incorporated into different *Leishmania* fatty acids varies, depending on the extent to which they are generated via *de novo* synthesis or scavenged from the media or host (S7 Fig., S8 Fig.). However, when rates of labeling were normalized to maximum labeling, similar rates of turnover were obtained regardless of the fatty acid measured. As expected, Pro^log^ exhibited fastest rates of fatty acid turnover, while both Pro^stat^ and Ama^axenic^ exhibited turnover rates that were comparable to, or slightly faster, than determined rates of protein turnover, respectively (Fig. 4). Strikingly, Ama^lesion^ exhibited very low rates of fatty acid turnover (t_1/2_ ~ 7.8 days, compared to 1.4 days in Ama^axenic^), indicating a dramatic slow-down in global rates of membrane biogenesis in lesion stages.

**Fig 5:**
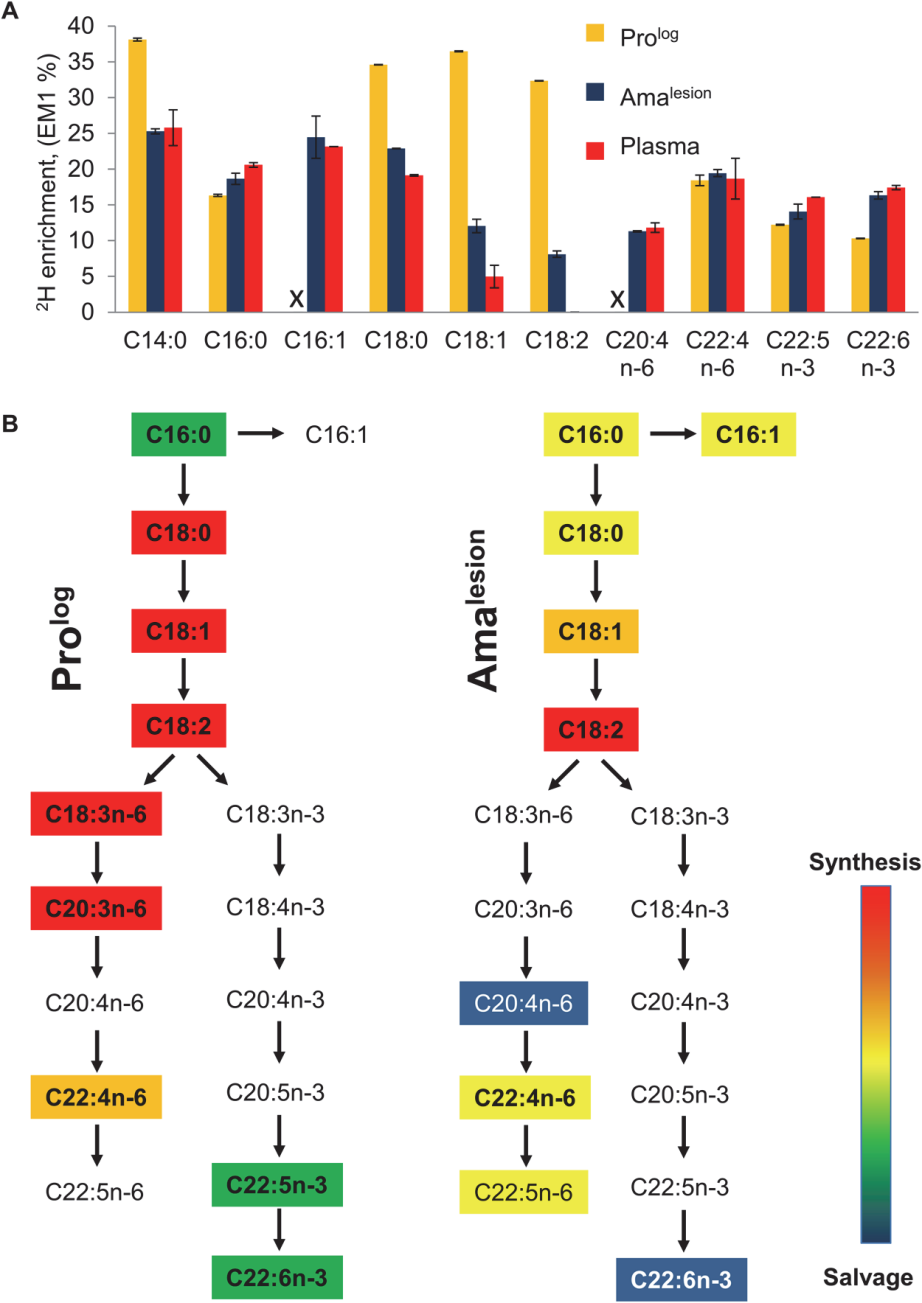
Lesion amastigotes utilize both salvage and *de novo* biosynthetic pathways to supply their fatty acid needs. **A**. The maximum level of ^2^H-enrichment (EM1, %) in major cellular fatty acids of Pro^log^ and Ama^lesion^ were determined after labeling for 7 days and >6 weeks, respectively. ^2^H-enrichment in the total plasma lipid of infected mice was also measured to determine the potential contribution of labeled host fatty acids to the parasite labeling. Note that while saturated and unsaturated C18 fatty acids are predominant fatty acids in both stages, the fatty acid composition of Ama^lesion^ differs from cultured promastigotes in containing elevated levels of C20:4 n-6, and polyunsaturated very long chain fatty acids (C22:4 n-6, C22:6 n-3) (S8 Fig.). The C:D nomenclature refers to overall chain length and number of double bonds in each fatty acid, respectively. n-3 and n-6 refers to the two major biosynthetic pathways involved in unsaturated fatty acid biosynthesis (where-3 and-6 refer to the position of double bond relative to the methyl carbon). **B**. Stage-specific differences in the levels of ^2^H-enrichment in fatty acid pools can be used to infer the contributions of *de novo* biosynthesis and salvage pathways. In particular, levels of ^2^H-enrichment in the major Ama^lesion^ C18 fatty acids, C18:1 (oleic acid) and C18:2 (linoleic acid), were appreciably higher than in host plasma, indicating that these stages are dependent on *de novo* biosynthesis. Conversely, the elevated levels of ^2^H-enrichment in C20:4 n-6 and C22:6n-3 compared to C18:1 precursor (comparable to plasma pools) indicate that these very long chain polyunsaturated fatty acids are primarily scavenged from the host cell.

To further investigate whether the ^2^H-labeling of Ama^lesion^ fatty acids reflects *de novo* synthesis or uptake of ^2^H-labeled fatty acids from the host [8,27], we measured the maximum levels of ^2^H-enrichment in both parasite and host fatty acids derived from plasma or lymph nodes after a prolonged period of labeling (Fig. 5, S9 Fig.). The labeling of C14:0, C16:0 and C16:1 fatty acids was comparable in both Ama^lesion^ and serum samples, and somewhat lower than maximum levels of enrichment in Pro^log^, indicating that lesion amastigotes are largely dependent on salvage pathways for these fatty acids. In contrast, maximum ^2^H-enrichment in Ama^lesion^ C18:0, C18:1 and C18:2 was higher than in equivalent host fatty acids. This was particularly pronounced for parasite C18:1 (oleic acid) and C18:2 (γ-linoleic acid) which were 2-fold or >50-fold more highly labeled than corresponding host fatty acids. The absence of ^2^H-enrichment in plasma C18:2 is consistent with the absence of Δ12 oleic acid desaturase in animals, while the elevated levels of labeling of amastigote C18:1 and C18:2 indicate that these fatty acid are predominantly synthesized by the parasite. Significantly, the rate of turnover of γ-linoleic acid was very similar to that Ama^lesion^ DNA providing additional support for the notion that these stages have a slow replication rate of ~12 days in vivo. Ama^lesion^ also contained higher levels of very long chain, polyunsaturated fatty acids, than cultured promastigotes (S8, S9 Figs). These included C20:4 n-6 (arachidonic acid) and C22:6 n-3 which were labeled to the same extent as the equivalent host fatty acids (Fig. 5A). Because the additional ^2^H-enrichment in parasite γ-linoleic acid is not observed in these downstream fatty acids, they are most likely salvaged directly from the host cell (Fig. 5B). Collectively, these results show that fatty acid/membrane turnover is dramatically reduced in lesion amastigotes. Notwithstanding their reduced requirements, these stages appear to be dependent on both salvage and *de novo* synthesis for maintaining fatty acid levels. In particular, our data suggest that they are likely to be critically dependent on the *de novo* synthesis of C18:2, which is not synthesized by the host and appears to be depleted in the macrophage phagolysosome compartment.

## Discussion

Information on host-parasite dynamics within *Leishmania*-induced lesions is limited, reflecting the technical difficulty of measuring the growth rate or physiological state of parasites within these tissues. Estimates of parasite growth based on direct enumeration of parasite numbers or detection of transgenic parasite lines expressing luciferase or fluorescent proteins are limited in sensitivity and do not distinguish between dynamic changes in the rate of pathogen replication, death or migration out of infected tissues. These approaches also require the generation of transgenic parasite lines which may alter virulence phenotypes and in which expression of reporter proteins may vary. In this study we have utilized ^2^H_2_O labeling to explicitly measure the growth rate and other key physiological parameters of wild type *L. mexicana* amastigotes in non-healing cutaneous lesions. We show that lesion amastigotes replicate very slowly (doubling time ~12 days) throughout lesion development and appear to enter a distinct semi-quiescent state, characterized by low rates of transcription, protein turnover and membrane biogenesis. Significantly, the calculated rates of amastigote growth account for the increase in the total parasite burden in isolated lesions, as well as the mean parasite densities in the phagolysosomes of lesion macrophages, suggesting that parasite death and turnover in these lesions occurs to a minimal extent. These analyses also provide direct evidence that infected macrophages in *L. mexicana*-induced granulomas have a long life span. Specifically, based on the average parasite burden of infected macrophages (100–200 amastigotes/ phagolysosome) it is likely that a significant majority of infected host cells in the lesion had been infected very early in the infection and have sustained intracellular parasites for >12 weeks. While it is possible that some macrophage death and turnover could be masked by direct transfer of parasite-laden vacuoles from a ruptured macrophage to a naive recipient host cell [28], these findings are broadly consistent with a growing body of evidence suggesting that *L. mexicana* amastigotes repress a number of signaling pathways in macrophages, including those that activate apoptosis, autophagy or necrosis [29,30]. Overall, these findings suggest that *L. mexicana*-induced lesions are characterized by slow parasite growth and low macrophage turnover. We propose that the slow replication rate of intracellular amastigotes (triggered in part by intrinsic amastigote differentiation signals), minimizes overgrowth of the phagolysosome and contributes to the long life-time of infected macrophages. Slow growth may therefore be a key factor in generating a stable, permissive tissue niche within which the parasite burden can progressively expand.

A number of other bacterial, fungal and protozoan parasites induce granulomatous structures in their host, of which the most intensively studied are the pulmonary granulomas induced by *Mycobacterium tuberculosis* [31,32]. Our findings suggest that host-pathogen dynamics in the *L. mexicana* induced lesions differ substantially from those in *M. tuberculosis* granulomas in several respects. In particular, the *M. tuberculosis*-induced granulomas are generally characterized by high rates of macrophage infiltration and host cell death [31,32]. There is also increasing evidence that bacterial replication and turnover in these granulomas may be relatively high as a result of host-mediated bacterial killing and/or immune clearance [6,12,32,33]. This contrasts with the earlier view that *M. tuberculosis* bacilli have a low replication rate, and corresponding low death rates, leading to the observed plateauing in bacterial numbers during chronic infections [6,12,32,33]. These observations highlight marked differences in the way different pathogens adapt to, and potentially exploit the host’s attempt to wall off persistent infection with granulomas. Whether these differences are defined by intrinsic differences in pathogen growth rate remain to be determined.

While a number of microbial pathogens are thought to switch to a quiescent or semi-quiescent state during long-term chronic phases of infection, relatively little is known about the physiological/metabolic state of these stages [3,4]. Here we show that slowly replicating lesion amastigotes strongly repress energy-expensive processes such as transcription, protein synthesis and membrane lipid turnover. The down-regulation of these processes in amastigotes was more pronounced than in non-dividing promastigotes, highlighting the fact that metabolic quiescence is not necessarily linked to growth rate and that non-dividing stages can remain metabolically active. It was notable, that repression of RNA synthesis was less pronounced than for either protein or fatty acid biosynthesis. *Leishmania* lack transcription factors and gene-specific transcriptional control [19] and a higher basal level of RNA turnover may be needed for post-transcriptional regulation of gene expression. The strong repression of protein synthesis in Ama^lesion^ (10-fold and 5-fold lower than in Pro^stat^ or Ama^axenic^, respectively) is consistent with recent studies demonstrating that promastigote to amastigote differentiation results in activation of the PERK kinase and phosphorylation of eIF2a, both of which regulate and repress protein translation and are required for amastigote virulence [34–36]. A general shut down in energy intensive processes, such as protein and fatty acid synthesis is also consistent with recent ^13^C-flux studies on isolated Ama^lesion^, which identified a unique stringent metabolic response in these stages, characterized by reduced carbon utilization and more efficient mitochondrial catabolism of sugars and fatty acids [24]. Thus the physiological responses of amastigotes to the lesion microenvironment are complex and regulated at the level of DNA replication, transcription and protein synthesis, in addition to remodeling of central carbon metabolism.

Entry into this semi-quiescent state is likely to be triggered by a number of factors. Both Ama^axenic^ and Ama^Mø^ exhibited slow maximum rates of growth (doubling time 3.5–4 days) and RNA turnover, suggesting that entry into this state is a hardwired response to elevated temperature and/or low pH used to induce differentiation *in vitro*. These findings also suggest that the phagolysosome of non-activated J774 macrophages are not restrictive for amastigote growth. On the other hand, there is evidence that the growth of *L. major* amastigotes in cutaneous lesions is restricted by the chronic production of sub-lethal levels of nitrous oxide [18]. However, *L. mexicana* amastigotes reside within larger communal phagolysosome compartments and are intrinsically more resistant to macrophage microbicidal processes, including nitric oxide or reactive oxygen species [14,37]. They also appear to have evolved additional mechanisms for inhibiting macrophage activation and nitric oxide production [38]. The slow growth rate of *L. mexicana* amastigotes in lesions may therefore reflect both intrinsic parasite responses, as well as adaptive responses to other host microbicidal processes, nutrient deprivation and/or physical stresses in this niche [24].

The high parasite inoculum used in our studies is expected to lead to rapid recruitment of macrophages and early induction of lesion development [39]. In contrast, infection of mice with a low dose inoculum is associated with a significant delay in lesion formation that is preceded by an exponential increase in parasite numbers [40]. In the *L. major*—C57BL murine model, this silent expansive phase is associated with an increase in parasite burden consistent with an apparent parasite doubling time of ~2.3 days [40]. This growth rate is similar to the replication rate we observed for *L. mexicana* amastigotes in non-activated J774 macrophages, raising the possibility that *Leishmania* amastigotes may switch between different growth states during acute and long-term chronic phases of infection. The possibility that amastigotes exhibit a range of growth rates within lesion was also suggested by the detection of a small number (<20%) of hyper-infected macrophages with more than 200 parasites/macrophage. The existence of distinct amastigote growth/physiological states is analogous to the situation in the sandfly vector. Initial colonization of the mid-gut of this host is mediated by ‘procyclic’ promastigotes that are rapidly dividing and generally more sensitive to a variety of physiological and nutritional stresses. These stages transition though a number of physiological states before differentiating to non-replicating, metacyclic promastigotes (related to Pro^stat^) in the foregut [41]. Metacyclic promastigotes are resistant to a number of stresses (including elevated temperature), suggesting that slow-growth represents a generalized response to elevated stresses in both the insect and mammalian hosts.

Many microbial pathogens acquire essential nutrients or metabolites via a combination of salvage pathways or *de novo* synthesis, leading to a level of redundancy that complicates efforts to identify and validate drug targets. While administration of ^2^H_2_O to infected mice results in metabolic labeling of both parasite and host lipids, analysis of the relative rates of labeling of parasite/host pools can be used to infer the contribution of salvage versus *de novo* biosynthetic pathway to parasite lipid homeostasis. In particular, we found that ^2^H-enrichment in Ama^lesion^ C18 fatty acids was substantially higher than in the equivalent host fatty acids, indicating that they are synthesize *de novo* by intracellular amastigotes. This was particularly striking for C18:2, which was labeled to a significant extent in Ama^lesion^, but unlabeled in mouse plasma samples. The absence of labeling of plasma C18:2 was not surprising given that animals lack the Δ12 desaturase needed to synthesize linoleic acid and are dependent on uptake of this fatty acid in the diet. The high maximum labeling of C18:2 in Ama^lesion^ indicates that this fatty acid is limiting for parasite growth in the phagolysosome compartment and therefore that amastigotes may be dependent on their own Δ12 desaturase for intracellular growth [42,43]. On the other hand, Ama^lesion^ contained elevated levels of very long chain, polyunsaturated fatty acids, including C20:4 n-6 (arachidonic acid), C22:4 n-6 (adrenic acid), C22:5 n-6 (osbond acid) and C22:6 n-3 (cervonic acid) compared to promastigotes. These fatty acids were ^2^H-enriched to a higher level than parasite C18 precursors fatty acids and were labeled to the same extent as the equivalent host fatty acids, suggesting that they are derived primarily via salvage pathways. These findings add to accumulating evidence that *Leishmania* amastigotes acquire a range of lipids from the host cell [24,44,45] and suggest that salvage as well as *de novo* biosynthesis pathways are potential drug targets.

In summary, we show that ^2^H_2_O labeling can be used as a universal labeling procedure to measure microbial growth, physiology and metabolism in their animal hosts. This approach is well suited for studying the *in vivo* growth and metabolism of other microbial pathogens.

## Materials and Methods

### Cell culture

*L. mexicana* promastigotes (Pro^log^, Pro^stat^) were cultured in RPMI 1640 medium supplemented with 10% (v/v) heat-inactivated fetal calf serum (FCS) at 27°C. Axenic amastigote (Ama^axenic^) were generated by adjusting the pH of the medium of stationary phase promastigotes (day 5, Pro^Stat^) to pH 5.5 with 1 M HCl and addition of 10% FCS (20% v/v final), followed by incubation at 33°C for four days.

### ^2^H_2_O labeling

Cultivated parasites stages (Pro^log^, Pro^stat^, Ama^axenic^) were cultured in medium supplemented directly with phosphate buffered saline- ^2^H_2_O (99.9%, Cambridge Isotopes) to give a final concentration of 5% (v/v) ^2^H_2_O.

J774 macrophages (4 × 10^6^) were grown overnight in RPMI 1640 medium supplemented with 10% (v/v) FCS, penicillin and streptomycin at 33°C [46], before being infected with *L. mexicana* Pro^stat^ at a MOI of 3. Non-internalized parasites were removed after 4 hr by washing macrophages twice with fresh RPMI medium. After 3 days, the medium of infected macrophages was replaced with fresh medium containing 5% ^2^H_2_O and cells harvested at various time points (ranging from 4 h and 16 days). For long term labeling experiments, the medium containing 5% ^2^H_2_O was replaced every 5 days.

BALB/c mice (6 week old) were infected with *L. mexicana* Pro^stat^ (10^6^ in 50 μl PBS) near the base of the tail and lesion size monitored as previously described [46]. Infected mice were injected intra-peritoneally with ^2^H_2_O (99%, 35 μl/g body weight) containing 0.9% NaCl after the development of nascent lesions and serum ^2^H_2_O concentration subsequently maintained at 5% by supplementation of the drinking water with 9% ^2^H_2_O. ^2^H_2_O levels in the urine were routinely monitored as previously described [47]. To determine maximum labeling parasite metabolites (EM_1_), mice were labeled with ^2^H_2_O immediately after the infection and parasites harvested one month after the development of a granulomatous lesion.

### Harvest of parasites

Cultured parasites stages were harvested with rapid metabolic quenching as previously described [48] and cell pellets (triplicate samples) were stored at -80°C prior to extraction. Infected J774 macrophages were metabolically quenched by chilling plates on ice and replacing the overlying culture medium with ice-chilled PBS. Infected macrophages were scraped from the plastic surface and lysed by repeated passage through a 25G needle (x10). After low speed centrifugation to remove host cell debris (60 g, 5 min, 4°C), amastigotes were recovered by sequential filtration through 5 μm and 3 μm pore filters and centrifugation (1500*g*, 10 min, 4°C) of the filtrate. The parasite pellet was washed three times with chilled PBS and any residual host cell DNA, removed by treatment of the pellet with 1000 U of DNAase in PBS with 5 mM MgCl_2_ for 2 h at 33°C.

Mice were culled humanely post-labeling (24 h to 150 days) and granulomatous lesions excised and immediately chilled in cold PBS. All subsequent procedures were carried out at <4°C. The isolated tissue mass was disrupted by passage through a cell strainer and host cells lyzed by passage through a 27 G syringe needle (x5). Intact host cells and host cell debris were removed by centrifugation (60 *g*, 10 min, 4°C) and released parasites harvested by centrifugation (1500 *g*, 10 min, 4°C). The pellet was washed three times with chilled PBS and the purity of the parasite extract confirmed by light microscopy. Samples for DNA analysis were treated with 1000 U of DNAase in PBS with 5 mM MgCl_2_ for 2 h at 33°C to remove any host cell DNA.

### Histology

BALB/c lesions (85 day post-infection) were fixed with 10% paraformaldehyde in PBS, embedded in paraffin and tissue sections stained with hematoxylin and eosin (H&E) reagents.

### Parasite extraction and sample preparation

Nucleic acids were extracted, hydrolyzed and dephosphorylated and released ribosyl and deoxyribosyl sugars derivitized as previously described, with some modifications [23,49,50]. In brief, nucleosides in 250μl H_2_O were incubated in HCl (0.01 M, 1.84 ml) and O-(2,3,4,5,6-pentafluorobenzyl) hydroxylamine acetate (PFBHA, 25 mg/ml, 20 μl), at 90°C for 3 h. Oximes of ribose and deoxyribose were extracted in ethyl acetate/hexane mix (1:1 v/v) followed by pure ethyl acetate and the pooled organic phases were dried under nitrogen. Samples were silylated by sequential addition of ethyl acetate (20 μl) and *N*,*O*-bis(trimethylsilyl) trifluoroacetamide reagent (40 μl BSTFA + 1% TMCS, Thermo scientific) and incubation at 90°C for 1 h. The perfluorotritrimethylsilyl (PFtriTMS) derivatives of deoxyribose and ribose were analyzed by GC/MS in negative and positive chemical ionization mode (NCI, PCI) with methane as reagent gas. The fragments at m/z 530 and m/z 633 are abundant PCI fragments of deoxyribose and ribose, respectively, that correspond to the loss of CH_4_: [M_+1_–16]^+^.

All derivitized samples were analyzed by GC/MS using a DB5 capillary column (J&W Scientific, 30 m, 250 μm inner diameter, 0.25 μm film thickness), with a 10 m inert duraguard. The injector insert and GC/MS transfer line temperatures were 270 and 250°C, respectively. The oven temperature gradient was set to: 70°C (1 min); 70°C to 295°C at 12.5°C/min;295°C to 320°C at 25°C/min; 320°C for 2 min. All metabolites were identified based on GC-retention times and mass spectra of standards. The excess M_1_ fraction and the fraction of new cells/molecules were calculated as described [51] following the measurement of the M_0_ and the corresponding M_+1_ ion using selected ion monitoring (SIM).

$$\text{Fraction of new molecules, f = }\frac{{\text{Enrichment (EM}}_{1})\text{, sample cells}}{{\text{Enrichment (EM}}_{1})\text{, fully turned over cells}}$$

$${\text{EM}}_{1}\text{ (Excess molar fraction) = }\frac{{{\text{(M}}_{+1}\text{ abundance)}}_{sample}}{{{\text{(M}}_{0}{\text{+ M}}_{+1}\text{ abundance)}}_{sample}}-\frac{{{\text{(M}}_{+1}\text{ abundance)}}_{unlab}}{{{\text{(M}}_{0}{\text{+ M}}_{+1}\text{ abundance)}}_{unlab}}$$

Parental ions (M_0_) were as follows: deoxyribose: PCI = 530 m/z, NCI = 525 m/z; ribose: PCI = 618 m/z, NCI = 452 m/z; alanine: PCI = 318 m/z; aspartate: PCI = 476 m/z; glutamate: PCI = 490 m/z; stearate: EI: 298 m/z, PCI = 327 m/z; oleate: EI = 296, PCI = 325 m/z; linoleate: EI = 294 m/z, PCI = 323 m/z. The half-life (t_½_) was determined by plotting data points on a log time (days) scale, fitting a straight line to the data points and solving the equation y = m*x + c for y = 0.5. The t½ is given by exp(log days).

Total lipids were extracted in chloroform/methanol/water (1:2:0.8 v/v) as described previously [48]. The organic phase was dried in a centrifugal evaporator and resuspended in chloroform/methanol (2:1 v/v, 30 μl) and total fatty acids analyzed as their methylesters after addition of Meth-Prep II reagent (5 μl, Grace Davison, Alltech) and direct injection and analysis by GC/MS in electron impact (EI) mode and positive chemical ionization mode. The delipidated protein pellets were hydrolyzed in 6 M HCl (200 μl, 110°C, 18 hr) and insoluble material removed by centrifugation (16,100 *g*, 5 min, RT). The supernatant was dried under nitrogen and released amino acids converted to their TBDMS derivatives by addition of MTBSTFA-1% TBDMCS (30 μl) and pyridine (30 μl, 60°C, 30 min) prior to GC-MS analysis in positive chemical ionization mode as described above.

### Enumeration of parasite numbers in lesion macrophages

BALB/c lesions (85 day post-infection) were fixed with 10% paraformaldehyde in PBS, embedded in paraffin and tissue sections stained with hematoxylin and eosin (H&E) reagents. Slides were imaged using a Zeiss Axioplan microscope and digital computer images were recorded with a Zeiss camera control unit and the corresponding dedicated software. The numbers of amastigotes was counted in 8 sections of 2 lesions evaluating >1200 phagolysosome in total. The number of parasites/vacuole was calculated assuming that (1) the phagolysosome compartment is a sphere, (2) that the average cross sectional diameter of the phagolysosome is equivalent to the largest vacuolar sections seen in the tissue sections and (3) that parasites are primarily arranged around the periphery of the phagolysosome at equal density. The average cross sectional diameter of the parasite occupied PVs was found to be 25.6 μm, while the average diameter of intracellular amastigotes was 2.5 μm.

To determine the maximum number parasites that fit in a vacuole section the ratio of the areas was calculated

$$\text{ratio of areas = }\frac{{\text{area}}_{vacuole}}{{\text{area}}_{parasite}}=104.9$$

and the maximum packing density of circles (PD_max2_) was calculated

$${\text{PD}}_{\max^{2}}\text{ = }\frac{\pi }{3\times \sqrt{2}}=0.90689$$

to estimate that the maximum number of parasites in a vacuole section (P_theor, max2_) is

$${\text{P}}_{theor,\max^{2}}\text{ = ratio of areas }\times {\text{PD}}_{\max^{2}}=95.1$$

Similarly, we determined the ratio of the volumes

$$\text{ratio of volumes = }\frac{{\text{volume}}_{vacuole}}{{\text{volume}}_{parasite}}=1073.7$$

and used maximum packing density of spheres (PD_max3_, Kepler conjecture)

$${\text{PD}}_{\max^{3}}\text{ = }\frac{\pi }{2\times \sqrt{3}}=0.74048$$

to determine that the maximum number of parasites in a vacuole (P_theor, max3_) is

$${\text{P}}_{theor,\max^{3}}\text{ = ratio of volumes }\times {\text{PD}}_{\max^{3}}=795.1$$

The number of parasites estimated to be present in the evaluated vacuoles (P_vac, estim_) was then determined based on the number of parasites counted in a vacuole section (P_count_) as follows

$${\text{P}}_{Vac,estim}\text{ = }\frac{{\text{P}}_{count}}{{\text{P}}_{theor,\max^{2}}}\times {\text{P}}_{theor,\max^{3}}$$

## Supporting Information

S1 Fig Metabolic pathways involved in ^2^H_2_O labeling of metabolites used in macromolecule synthesis. In the presence of 5% ^2^H_2_O, deuterium is enzymatically incorporated (reversible red arrows) into ribose and deoxyribose nucleotides via the pentose phosphate pathway, various hexose-phosphate isomerization reactions and ribonucleotide reductase. Label can also be incorporated into hexose phosphates via gluconeogenesis. Deuterium label is incorporated into amino acids and fatty acids via multiple pathways including transamination reactions, the tricarboxylic acid cycle and fatty acid synthases.
(TIFF)

S2 Fig Salvage of pentose sugars, nucleoside phosphates and deoxyribonucleosides is negligible. The maximum ^2^H enrichment in promastigotes DNA deoxyribose was measured for parasites grown in RPMI 1640 containing 5% ^2^H_2_O supplemented with pentose sugars, nucleoside phosphates and deoxyribunucleosides (each at 7 mM). Addition of these putative dRib precursors had little effect on parasite growth or labeling of the dRib in parasite DNA. Maximum decrease was 15% (deoxycytidine) relative to parasites grown in non-supplemented media. These data suggest that ribose salvage pathways do not contribute significantly to DNA synthesis, and therefore that incorporation of ^2^H-into DNA dRib provides an accurate reflection of DNA synthesis.
(TIFF)

S3 Fig A stable concentration of ^2^H_2_O is maintained in the body water of mice. Mice were given a bolus of ^2^H_2_O and were subsequently fed 9% ^2^H_2_O in the drinking. This regime led to a stable ^2^H_2_O concentration of 5% (v/v) in the mouse body water for several weeks to months.
(TIFF)

S4 Fig Histology of *L. mexicana* induced lesions in BALB/c mice. Lesions were excised from BALB/c mice and sections stained with Hematoxylin and Eosin (H&E). Montage of light microscope images of the lesion and detail (x 60 magnification) are shown. The lesions were composed primarily of heavily infected host cells containing large communal parasite-induced vacuoles.
(TIFF)

S5 Fig Contaminating DNA can be removed from isolated parasites using DNase treatment. **A**. Bright field image of Ama^lesion^ preparation showing absence of significant contamination with host cells and/or nuclei. **B**. Ama^lesion^ released from murine lesions contain host DNA that is carried over from lysed host cells. To confirm that the level of contaminating host DNA is low and largely removed by DNase treatment, we quantitated total DNA and extracellular DNA in the lesion preparations (pre- and post-DNase treatment). DNA levels were determined by spectrophotometric measurement of DAPI fluorescence (fluorescence units) (error bars are for two technical replicates). These analyses show that contaminating host DNA accounts for less than 20% of the total parasite DNA after DNase treatment. **C**. To further confirm that DNase treatment effectively removes contaminating extracellular DNA, Ama^lesion^ were metabolically labeled with ^13^C-glucose (to incorporate uniformly labeled dRib into the DNA) and preparations spiked with excess unlabeled salmon sperm DNA (5-fold over parasite DNA). The level of contamination with salmon DNA was subsequently determined by GC-MS analysis of DNA-dRib and quantitation of the ratio of M_+5_/M_0_ isotopomers. Pretreatment of live parasites with DNase restored the M_+5_/M_0_ ratio of the DNA-deoxyribose to that found in parasites which had not be exposed to extracellular DNA, confirming that DNase effectively removes contaminating DNA.
(TIFF)

S6 Fig Similar protein turnover rates are calculated from the labeling information of different proteinogenic amino acids. Infected BALB/c mice were labeled with ^2^H_2_O for the indicated time points and incorporation of deuterium into isolated Ama^lesion^ proteinogenic amino acids determined by GC-MS. **A**. Maximum enrichment achieved in the proteinogenic amino acids, alanine, aspartate and glutamate, were ~ 11, 4 and 9%, EM_1_, respectively. **B**. Similar protein turnover times were calculated based on the labeling kinetics of these amino acids are (turnover times (in days) are given in insert box). Because of the high maximum ^2^H-labeling of alanine, the ^2^H-enrichment in this amino acid was routinely used to measure protein turnover times.
(TIFF)

S7 Fig Kinetics of ^2^H-labeling of major C18 fatty acids in Ama^lesion^. **A**. Infected BALB/c mice were labeled with ^2^H_2_O for the indicated time points and incorporation of deuterium into isolated Ama^lesion^ total cellular fatty acids determined as their methyl esters by GC-MS. **B**. Differences in the maximum level of deuterium enrichment were observed reflecting differences in the contribution of parasite and host fatty acid biosynthetic/salvage pathways to the bulk Ama^lesion^ composition. Unlike stearic and oleic acid, linoleic acid is exclusively synthesized by the parasite and rates of turnover of this fatty acid reflect rates of parasite replication determined by analysis of deuterium incorporation into d-Rib DNA.
(TIFF)

S8 Fig Fatty acid composition of Pro^log^ and Ama^lesion^. Lipids were extracted from (**A**) Pro^log^ and (**B**) purified Ama^lesion^ in chloroform/methanol/water and total fatty acids determined after methyl-esterification and GC-MS. The major fatty acids in both stages were C18 fatty acids. Details of GC-MS chromatograms highlighting differences in the relative abundance of C18-C20 fatty acids and C22 fatty acids are shown in panels **C** and **D**, respectively. The C:D nomenclature refers to overall carbon chain length and number of double bonds in each fatty acid, respectively. n-3 and n-6 refers to the two major biosynthetic pathways involved in unsaturated fatty acid biosynthesis (where-3 and-6 refer to the position of double bond relative to the methyl carbon).
(TIFF)

S9 Fig Fatty acid composition of mouse plasma and lymph nodes. Total cellular lipids were extracted from (**A**) plasma and (**B**) inguinal lymph nodes of *L. mexicana*-infected mice and the fatty acid composition determined by GC-MS after methyl-esterification. **C**. Detail of GC-MS chromatograms showing differences in C22 poly-unsaturated fatty acids between the two tissues.
(TIFF)
