# Supplementary figures and images for: Characterization of Metabolically Quiescent Leishmania Parasites in Murine Lesions Using Heavy Water Labeling
Source: PLoS Pathog. 2015 Feb 25;11(2):e1004683. doi: 10.1371/journal.ppat.1004683 (PMC4340956; doi:10.1371/journal.ppat.1004683)

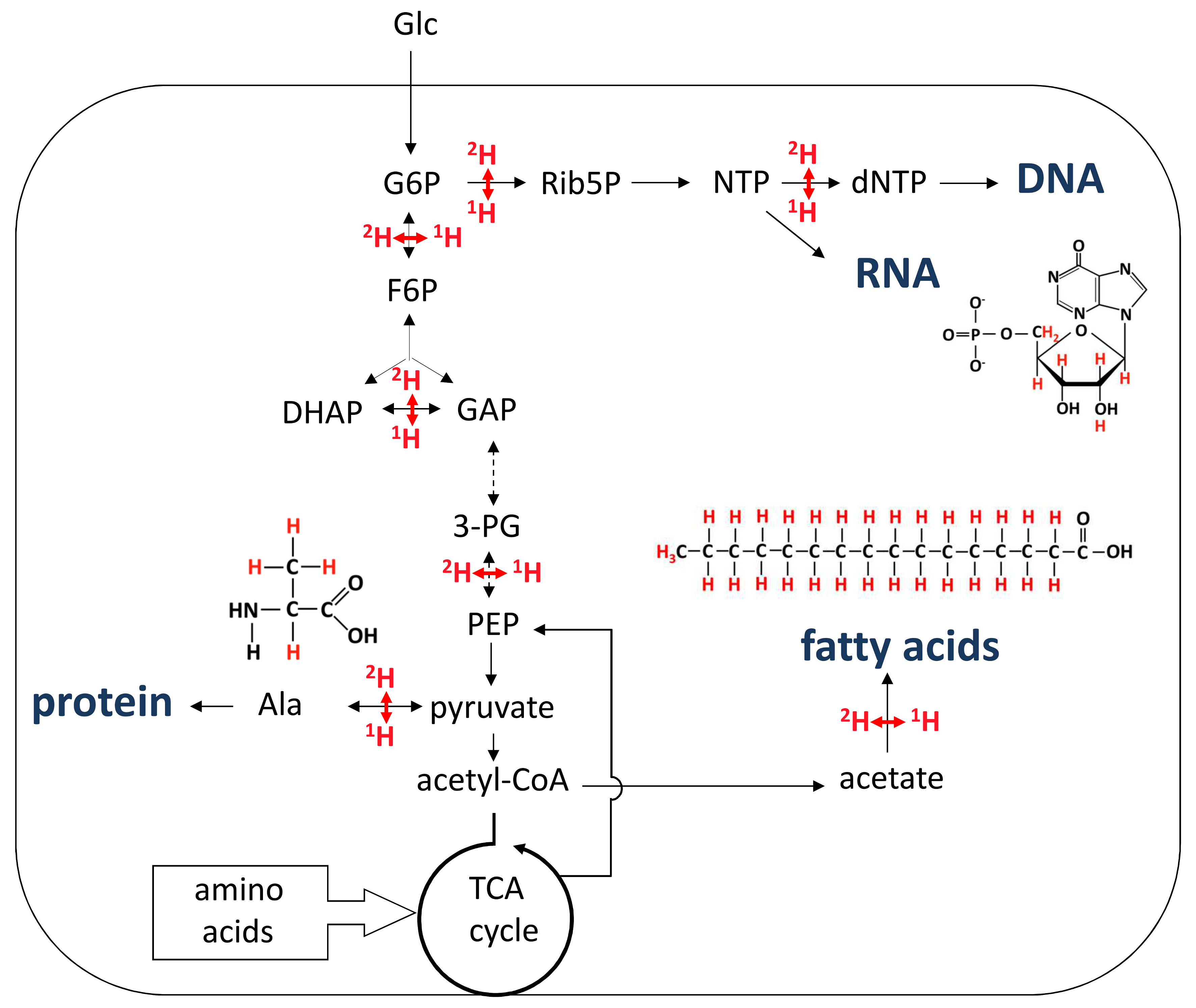

Supplement: S1 Fig — In the presence of 5% 2H2O, deuterium is enzymatically incorporated (reversible red arrows) into ribose and deoxyribose nucleotides via the pentose phosphate pathway, various hexose-phosphate isomerization reactions and ribonucleotide reductase. Label can also be incorporated into hexose phosphates via gluconeogenesis. Deuterium label is incorporated into amino acids and fatty acids via multiple pathways including transamination reactions, the tricarboxylic acid cycle and fatty acid synthases. (TIFF) [file ppat.1004683.s001.tiff]

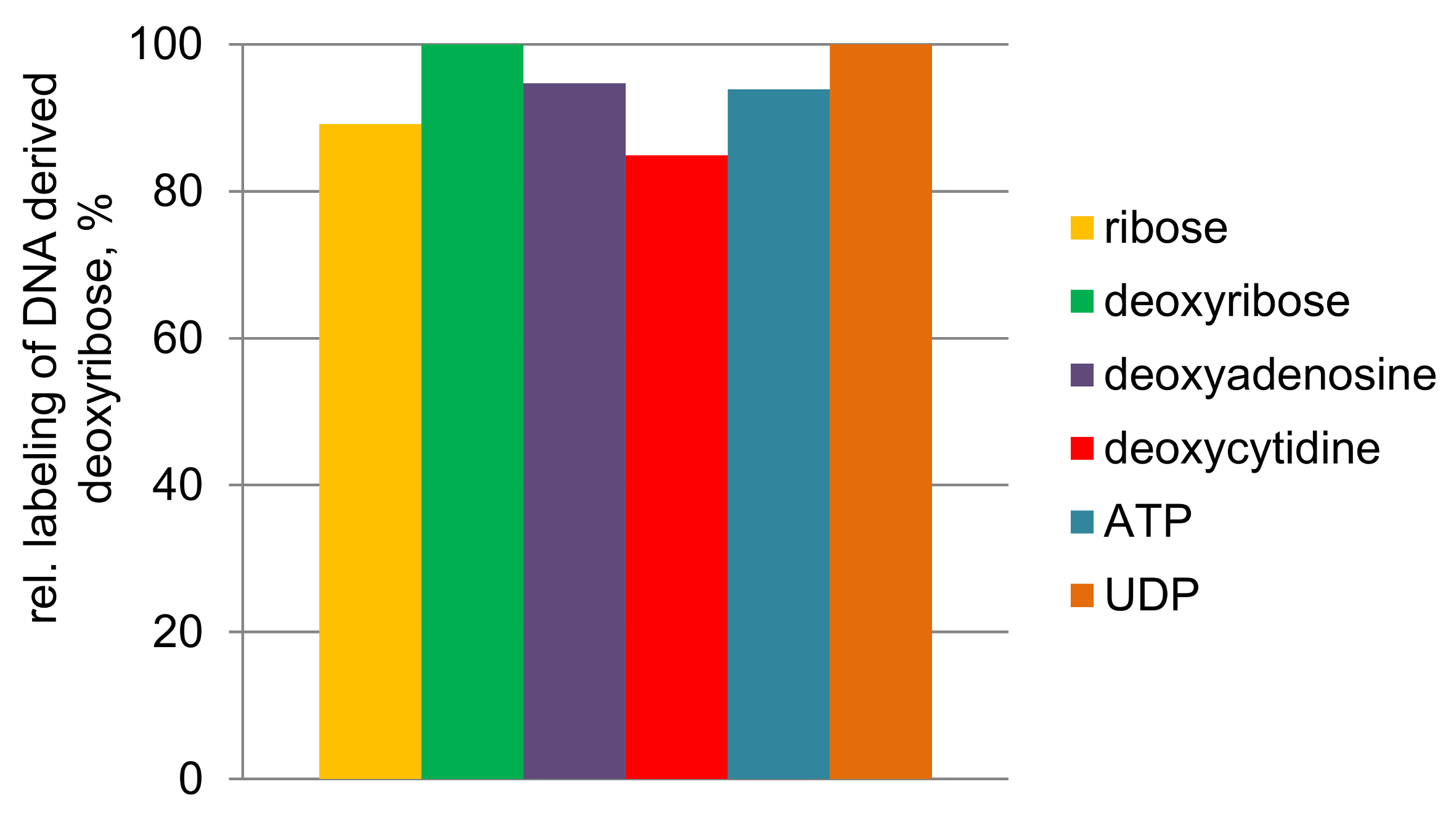

Supplement: S2 Fig — The maximum 2H enrichment in promastigotes DNA deoxyribose was measured for parasites grown in RPMI 1640 containing 5% 2H2O supplemented with pentose sugars, nucleoside phosphates and deoxyribunucleosides (each at 7 mM). Addition of these putative dRib precursors had little effect on parasite growth or labeling of the dRib in parasite DNA. Maximum decrease was 15% (deoxycytidine) relative to parasites grown in non-supplemented media. These data suggest that ribose salvage pathways do not contribute significantly to DNA synthesis, and therefore that incorporation of 2H-into DNA dRib provides an accurate reflection of DNA synthesis. (TIFF) [file ppat.1004683.s002.tiff]

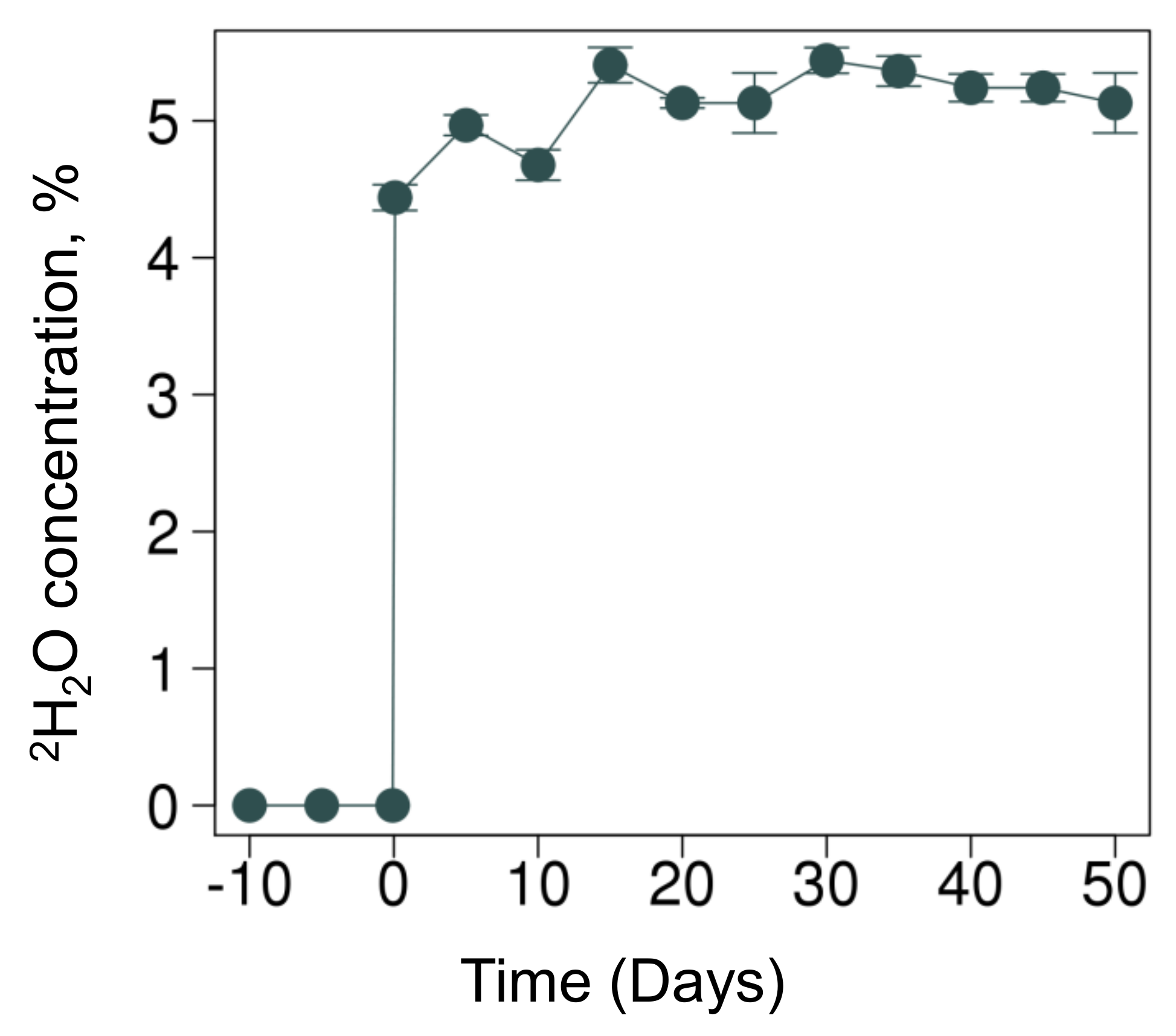

Supplement: S3 Fig — Mice were given a bolus of 2H2O and were subsequently fed 9% 2H2O in the drinking. This regime led to a stable 2H2O concentration of 5% (v/v) in the mouse body water for several weeks to months. (TIFF) [file ppat.1004683.s003.tiff]

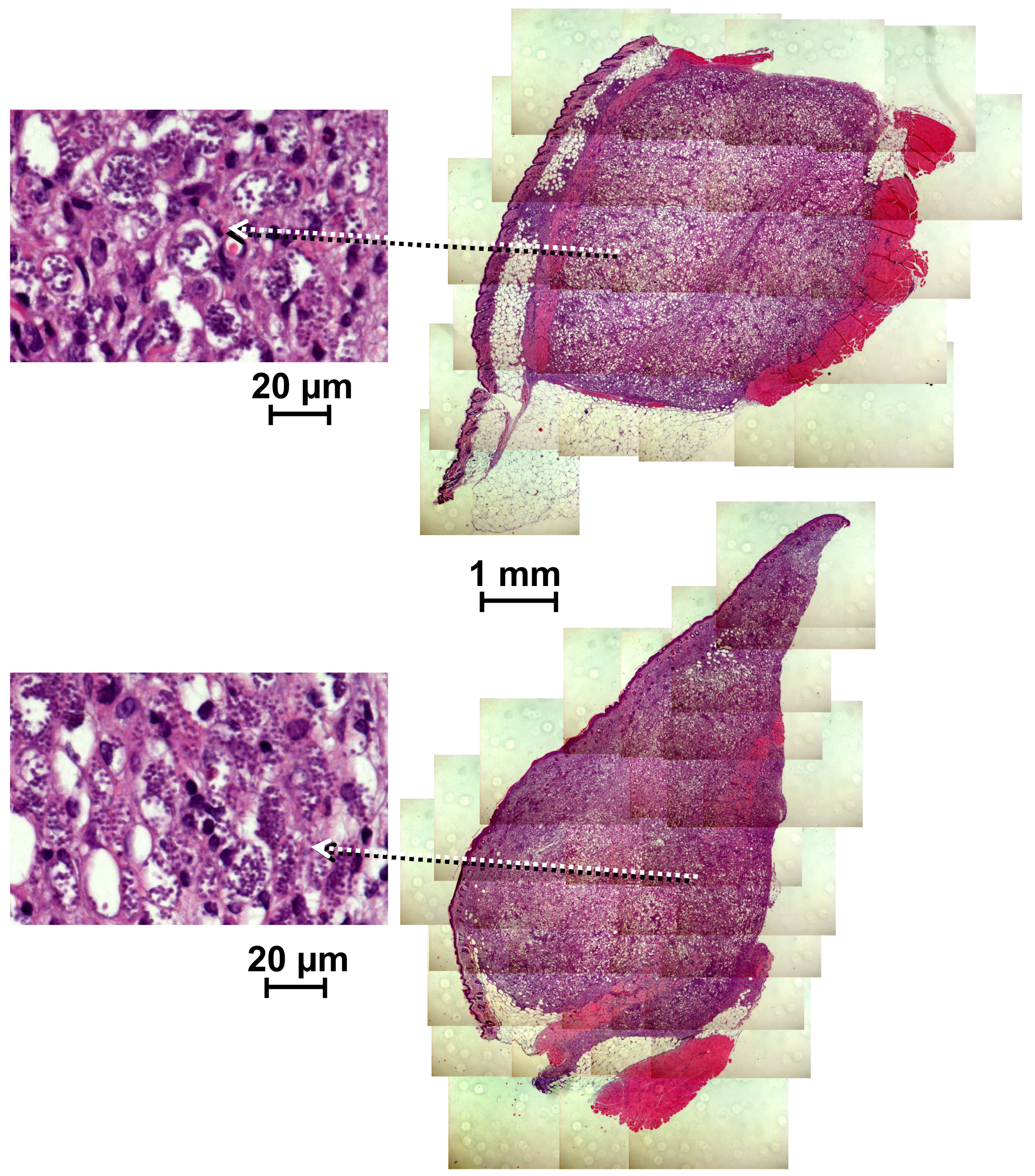

Supplement: S4 Fig — Lesions were excised from BALB/c mice and sections stained with Hematoxylin and Eosin (H&E). Montage of light microscope images of the lesion and detail (x 60 magnification) are shown. The lesions were composed primarily of heavily infected host cells containing large communal parasite-induced vacuoles. (TIFF) [file ppat.1004683.s004.tiff]

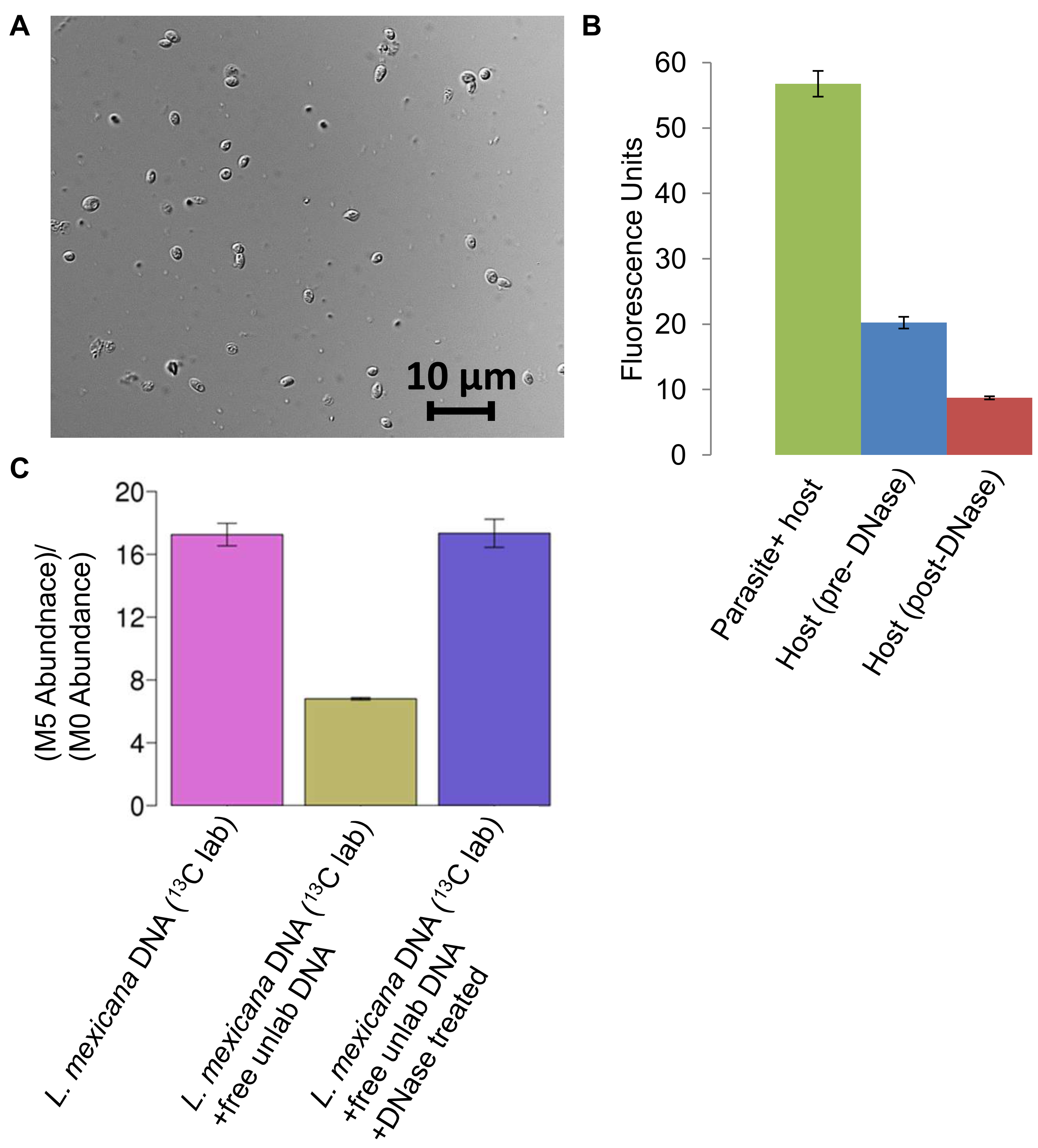

Supplement: S5 Fig — A. Bright field image of Amalesion preparation showing absence of significant contamination with host cells and/or nuclei. B. Amalesion released from murine lesions contain host DNA that is carried over from lysed host cells. To confirm that the level of contaminating host DNA is low and largely removed by DNase treatment, we quantitated total DNA and extracellular DNA in the lesion preparations (pre- and post-DNase treatment). DNA levels were determined by spectrophotometric measurement of DAPI fluorescence (fluorescence units) (error bars are for two technical replicates). These analyses show that contaminating host DNA accounts for less than 20% of the total parasite DNA after DNase treatment. C. To further confirm that DNase treatment effectively removes contaminating extracellular DNA, Amalesion were metabolically labeled with 13C-glucose (to incorporate uniformly labeled dRib into the DNA) and preparations spiked with excess unlabeled salmon sperm DNA (5-fold over parasite DNA). The level of contamination with salmon DNA was subsequently determined by GC-MS analysis of DNA-dRib and quantitation of the ratio of M+5/M0 isotopomers. Pretreatment of live parasites with DNase restored the M+5/M0 ratio of the DNA-deoxyribose to that found in parasites which had not be exposed to extracellular DNA, confirming that DNase effectively removes contaminating DNA. (TIFF) [file ppat.1004683.s005.tiff]

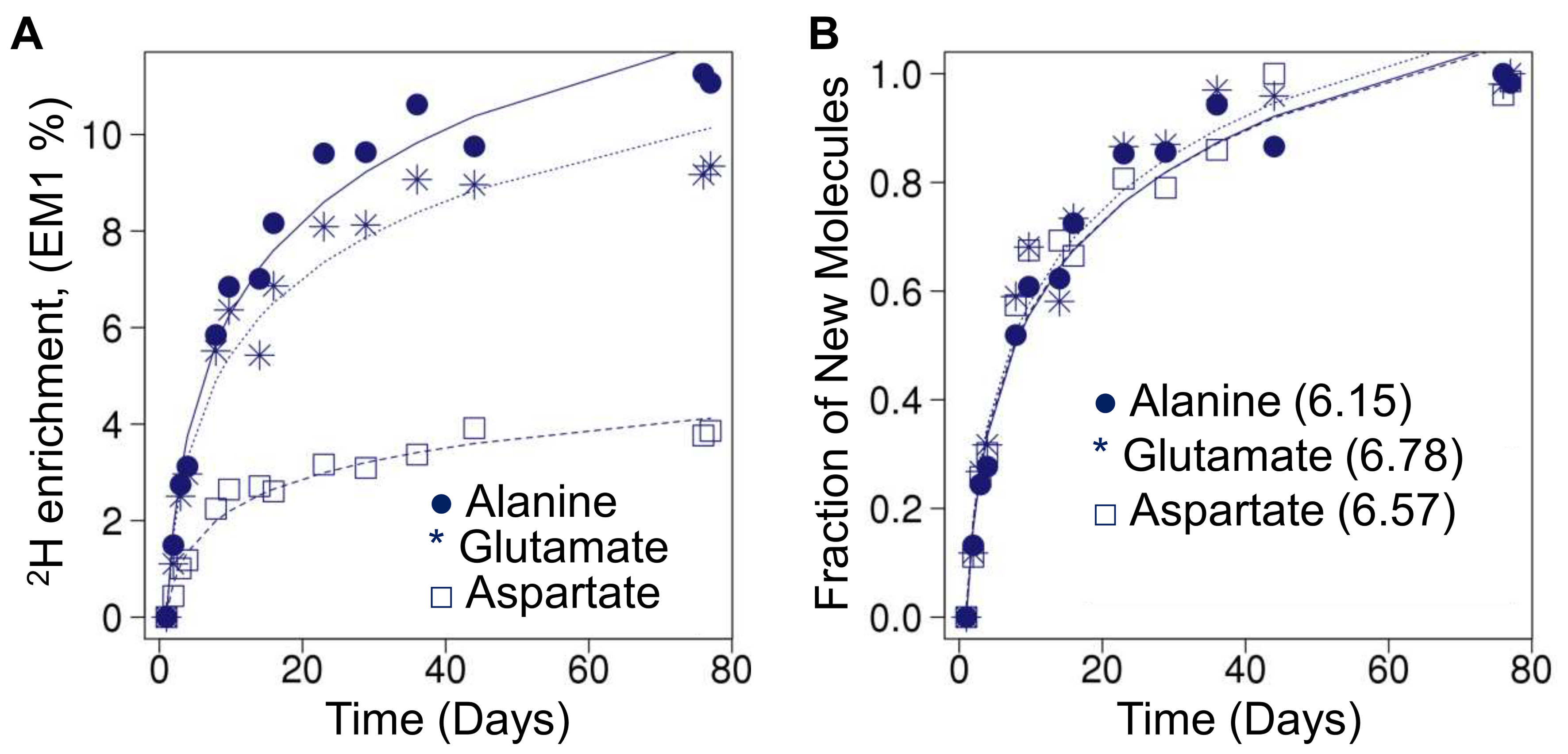

Supplement: S6 Fig — Infected BALB/c mice were labeled with 2H2O for the indicated time points and incorporation of deuterium into isolated Amalesion proteinogenic amino acids determined by GC-MS. A. Maximum enrichment achieved in the proteinogenic amino acids, alanine, aspartate and glutamate, were ~ 11, 4 and 9%, EM1, respectively. B. Similar protein turnover times were calculated based on the labeling kinetics of these amino acids are (turnover times (in days) are given in insert box). Because of the high maximum 2H-labeling of alanine, the 2H-enrichment in this amino acid was routinely used to measure protein turnover times. (TIFF) [file ppat.1004683.s006.tiff]

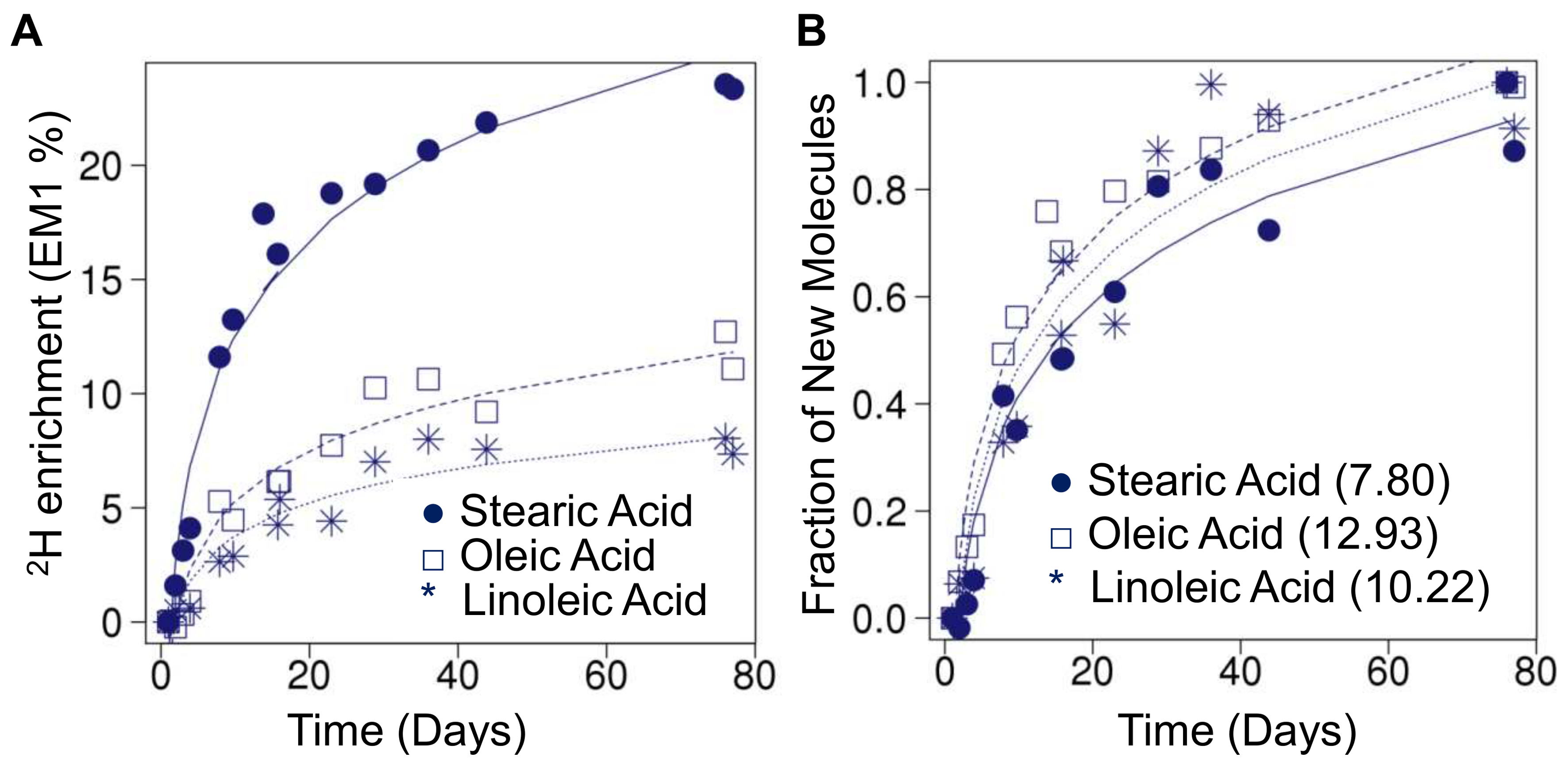

Supplement: S7 Fig — A. Infected BALB/c mice were labeled with 2H2O for the indicated time points and incorporation of deuterium into isolated Amalesion total cellular fatty acids determined as their methyl esters by GC-MS. B. Differences in the maximum level of deuterium enrichment were observed reflecting differences in the contribution of parasite and host fatty acid biosynthetic/salvage pathways to the bulk Amalesion composition. Unlike stearic and oleic acid, linoleic acid is exclusively synthesized by the parasite and rates of turnover of this fatty acid reflect rates of parasite replication determined by analysis of deuterium incorporation into d-Rib DNA. (TIFF) [file ppat.1004683.s007.tiff]

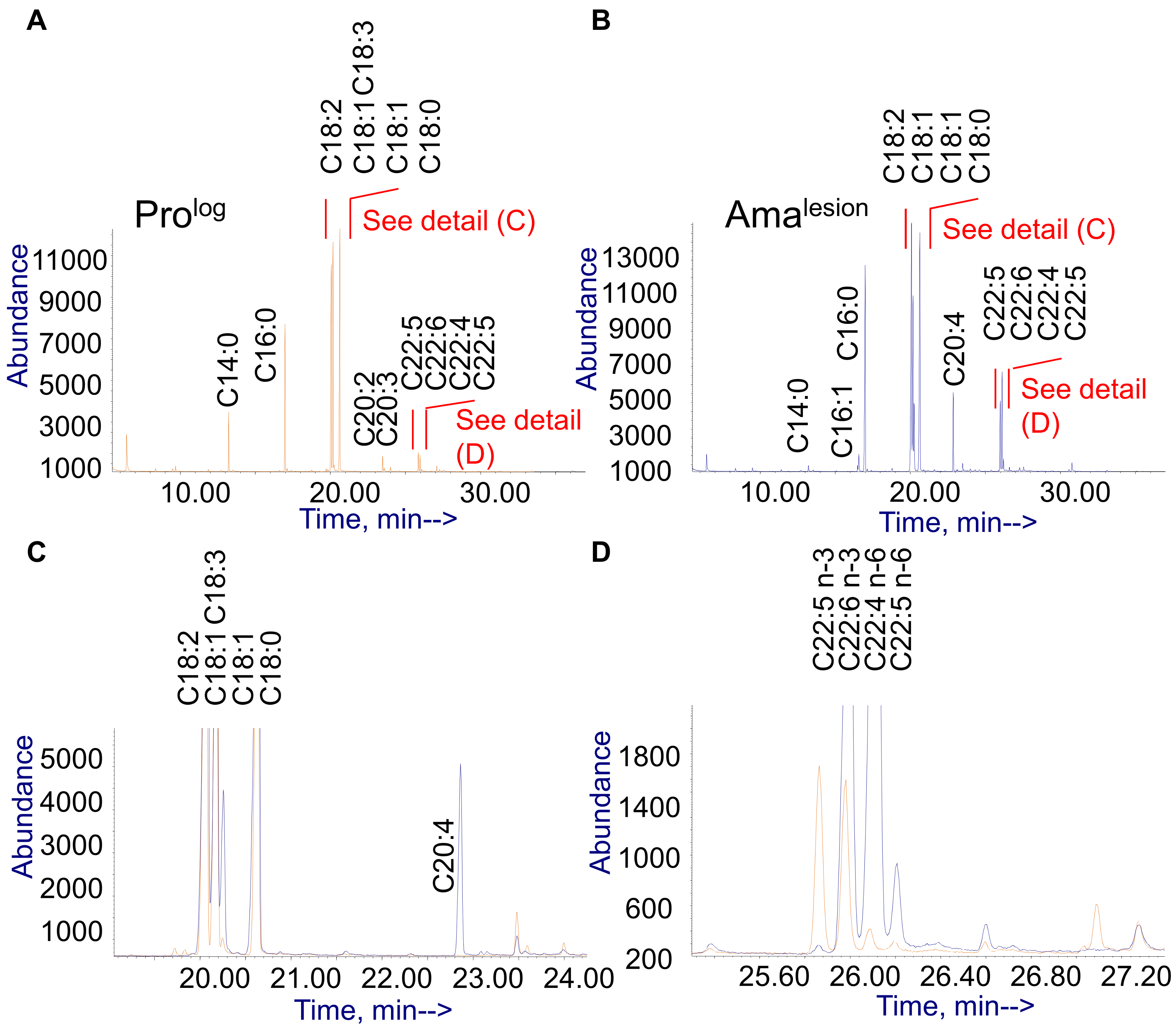

Supplement: S8 Fig — Lipids were extracted from (A) Prolog and (B) purified Amalesion in chloroform/methanol/water and total fatty acids determined after methyl-esterification and GC-MS. The major fatty acids in both stages were C18 fatty acids. Details of GC-MS chromatograms highlighting differences in the relative abundance of C18-C20 fatty acids and C22 fatty acids are shown in panels C and D, respectively. The C:D nomenclature refers to overall carbon chain length and number of double bonds in each fatty acid, respectively. n-3 and n-6 refers to the two major biosynthetic pathways involved in unsaturated fatty acid biosynthesis (where-3 and-6 refer to the position of double bond relative to the methyl carbon). (TIFF) [file ppat.1004683.s008.tiff]

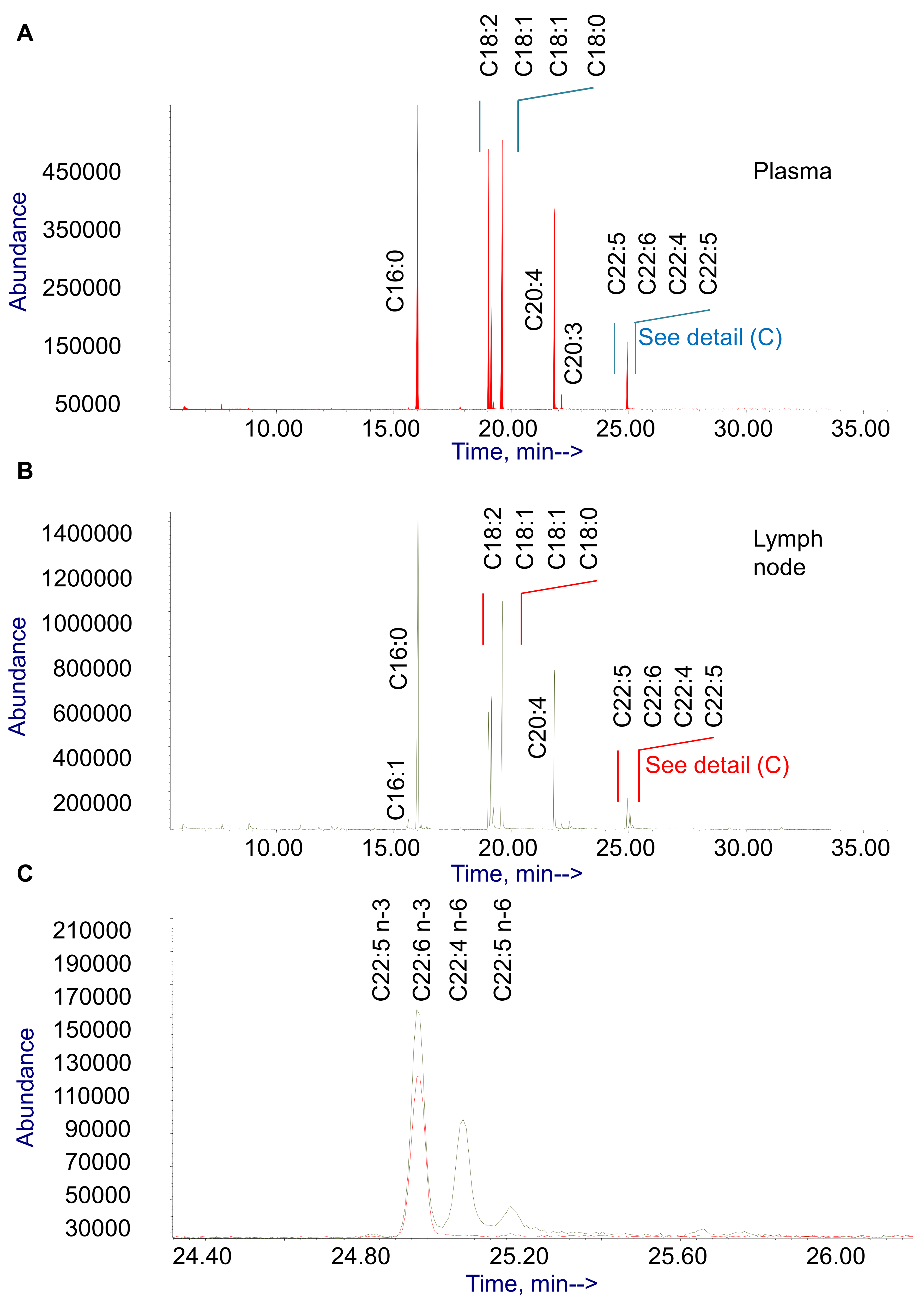

Supplement: S9 Fig — Total cellular lipids were extracted from (A) plasma and (B) inguinal lymph nodes of L. mexicana-infected mice and the fatty acid composition determined by GC-MS after methyl-esterification. C. Detail of GC-MS chromatograms showing differences in C22 poly-unsaturated fatty acids between the two tissues. (TIFF) [file ppat.1004683.s009.tiff]
